# Tape Stripping Method in Dermatological and Pharmacological Research: Evaluating Fresh and Frozen-Thawed Porcine Skin

**DOI:** 10.7759/cureus.68477

**Published:** 2024-09-02

**Authors:** Dylan Rowe, Mariam Rowe, Chase Pontifex, Dylan Stubbs

**Affiliations:** 1 School of Medicine, Griffith University, Brisbane, AUS; 2 Faculty of Medicine, University of Queensland, Brisbane, AUS; 3 School of Medicine, Griffith University, Gold Coast, AUS

**Keywords:** histology, skin specimen preservation, topical treatments, dermatological research, fresh versus frozen skin, tape weighing, skin barrier function, porcine skin, tape stripping, stratum corneum

## Abstract

Background

The stratum corneum (SC) plays a crucial role in protecting the skin and regulating water loss. Tape stripping is a well-established method for studying skin barrier function and evaluating topical treatments. However, the behavior of fresh versus frozen-thawed skin during tape stripping has not been extensively compared.

Objective

This study aims to compare the removal of the stratum corneum from fresh and frozen-thawed porcine skin using tape stripping. It also aims to assess the reliability of tape weighing versus histological methods in quantifying SC removal.

Methods

Fresh and frozen-thawed porcine ears were obtained, cleaned, and subjected to tape stripping at varying numbers of strips from zero to 40. Tape weight and histological measurements were used to quantify SC removal. Statistical analyses were conducted to compare SC thickness and tape weight between the two types of skin.

Results

The study found that frozen-thawed skin exhibited a non-linear rate (r = 0.65) of SC removal per tape strip in the first five strips compared to a linear removal for fresh skin (r = 0.96). By the fifth tape strip, frozen-thawed samples had lost 80.6% of their SC, while fresh samples had only lost 33.5% (P < 0.03). Tape weighing and histological measurements showed strong correlations (r = 0.93 for fresh skin and r = 0.95 for frozen-thawed skin), indicating that tape weighing is a reliable alternative to histology for assessing SC removal on both sample types.

Conclusions

Fresh and frozen-thawed porcine skin respond differently to tape stripping, with frozen-thawed skin showing accelerated SC removal in the first five strips. The strong correlation between tape weighing and histological analysis supports the use of tape weighing as a practical tool for evaluating SC removal. These findings have implications for specimen selection and methodological standardization in dermatological and pharmacological research. Future research should explore alternative preservation and SC thickness measurement methods and their impact on tape stripping outcomes.

## Introduction

The stratum corneum (SC), the outermost layer of the skin, is vital for protecting against environmental hazards and regulating water loss [1]. Gaining insights into its properties and responses to various treatments is crucial for advancing dermatological research and clinical practice. Tape stripping, a method first described in 1939 [2], has become a key technique for studying skin barrier function, drug penetration, and the efficacy of topical formulations [3-5]. Compared to skin biopsies, tape stripping is less invasive and applicable in both ex vivo and in vivo studies [6].

Accurately quantifying the amount of stratum corneum removed by each tape strip is essential for the validity of tape stripping studies. Various methods have been employed to achieve this, including microscopy [7], transepidermal water loss (TEWL) measurements [8,9], infrared spectroscopy [10,11], infrared densitometry [12], electrical impedance spectroscopy [13], and protein content analysis of removed tape strips [14-16]. In skin penetration studies, porcine skin is often used as a substitute for human skin due to its similar permeation characteristics, including lag time, diffusion behavior, and SC thickness [17-21]. These similarities suggest that findings from porcine skin studies can be transferable to human skin.

Frozen-thawed skin is commonly used for testing because it is more accessible than fresh skin samples. However, previous research has shown that the structural integrity and cellular composition of the stratum corneum can be altered by freezing and thawing processes [22]. Studies have reported differing responses to tape stripping when comparing fresh and frozen-thawed samples. For instance, histological measurements have shown that frozen-thawed skin exhibits a linear decrease in SC thickness with successive tape strips [23]. Other studies using methods such as skin hydration, pH, and TEWL have found contrasting results [24], highlighting the need for further investigation using a consistent measurement technique across both types of skin samples.

This study aims to contribute to the ongoing research by examining the differences in the behavior of fresh and frozen-thawed porcine skin during tape stripping. Unlike previous studies that focused solely on the cells removed or only examined one type of sample (fresh or frozen-thawed), we quantified both the amount of cells removed and the thickness of the SC left behind at successive points throughout the tape stripping process using tape weighing and histological methods. By comparing the responses of fresh and frozen-thawed porcine specimens to tape stripping, we seek to inform specimen selection, improve consistency in experimental protocols, and enhance the interpretation of results in studies involving skin barrier function and topical treatments. Additionally, this study compares the reliability of tape weighing and histological methods for assessing SC removal across the two skin sample types, providing valuable insights for future research.

## Materials and methods

Porcine skin

Animal experiments were carried out in our institution's testing laboratory in accordance with the regulations set by the Australian Animal Ethics Committee. Ethical approval from the committee was not necessary as the samples were sourced from the local abattoir's food chain. For this study, six fresh porcine ears were obtained from a local abattoir. Three ears were tested fresh within five hours of the animals' slaughter, while the other three ears were frozen within five hours of collection and stored at -20°C until needed. Before testing, the frozen ears were thawed at room temperature for four hours, where the testing laboratory was environmentally controlled using standard protocols (24 ± 1°C and 40 ± 5% RH). All samples were cleaned with 70% isopropyl alcohol to remove visible contaminants and dried with a paper towel, and any hair was removed using an electric shaver.

Tape stripping

The established tape stripping method [2,7] was employed to selectively remove layers of cells from the stratum corneum. Nine 2 cm² test sites, visually similar in color and texture, were chosen per ear specimen and marked with a permanent marker to ensure precise and consistent tape application. Each test site had a varying number of tape applications: 0 (baseline), 1, 2, 3, 4, 5, 10, 20, and 40. This sequence was repeated for each of the three fresh and three frozen-thawed porcine ears, equating to a total of 54 test sites across the six specimens. A fresh section of clear cellophane tape was cut to 2 cm^2^ for each application. The tape was applied to the test site, held in place at 5 N for three seconds, and then removed in one swift motion. Each subsequent piece of tape was applied after rotating the specimen 90 degrees. The final test site used a total of 40 tape strips to ensure the complete removal of the stratum corneum [23]. The effectiveness of the stripping was also assessed by visually inspecting the site for a shiny, wet appearance, as described by Lindemann et al. [7].

Precision tape weighing

Each section of clear cellophane tape was cut and weighed on the ISG 153-104 precision balance scale (International Scientific, Peterborough, United Kingdom), and the tapes' pre-test weight was recorded. The tape was applied to the test site and held in place at 5 N for three seconds before being removed in one swift movement. The tape was then weighed on the ISG precision balance scale, and the post-test weight was recorded.

Histology

Each test site was biopsied and fixed in formalin for 24 hours before being transferred to 70% ethanol for transport. Paraffin fixation was employed in the histology laboratory, followed by standard automated hematoxylin and eosin (H&E) staining. The 2 cm² square samples were sliced through their centers, and single slide images were digitally scanned for each sample site. CaseViewer (3DHISTECH, Budapest, Hungary) software was utilized to analyze these images and measure the thickness of the stratum corneum at each test site. These measurements facilitated the correlation of penetration depth with the number of tape strips applied.

Statistical analysis

The data was tabulated and analyzed to determine the mean and standard deviation (SD), and all results are given as mean ± SD. An unpaired t-test was used to assess the difference in skin thickness after tape stripping between the fresh and frozen-thawed samples where statistical significance was set at P < 0.05. Pearson correlation coefficient was used to compare the tape weighing method to the histological thickness measurements for both fresh and frozen-thawed samples, where a strong correlation was set at r > 0.80 (or less than -0.80).

## Results

The amount of stratum corneum removed at each test site after tape stripping was determined by two separate methods. First, the thickness of the remaining stratum corneum on the porcine skin specimen was digitally measured on the scanned H&E slide image. Second, the difference in weight of the tape pre-application and post-application was compared across all test sites for each tape strip.

Stratum corneum thickness

**Figure 1:**
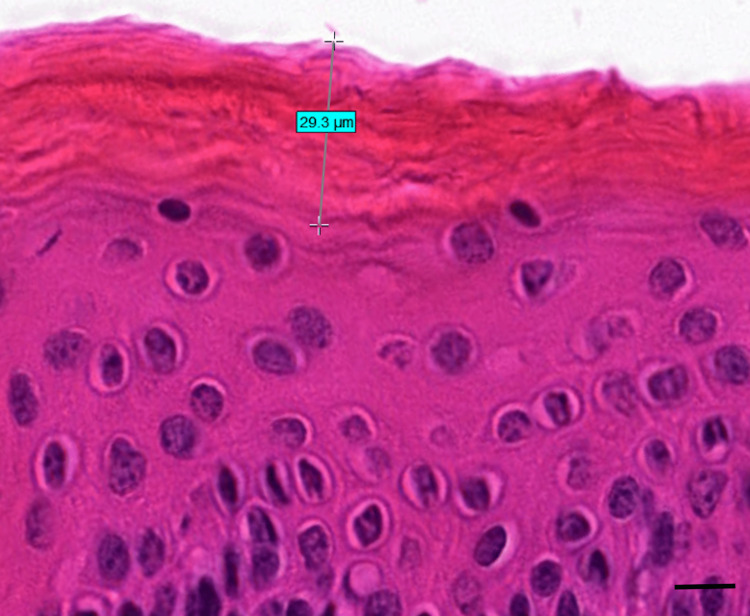
Histological slide of the pre-stripped skin with full stratum corneum visible (baseline) (H&E stain) The stratum corneum thickness is 29.3 µm. This sample was taken from the frozen-thawed porcine skin. The scale bar represents 10 µm. H&E: hematoxylin and eosin

**Figure 2:**
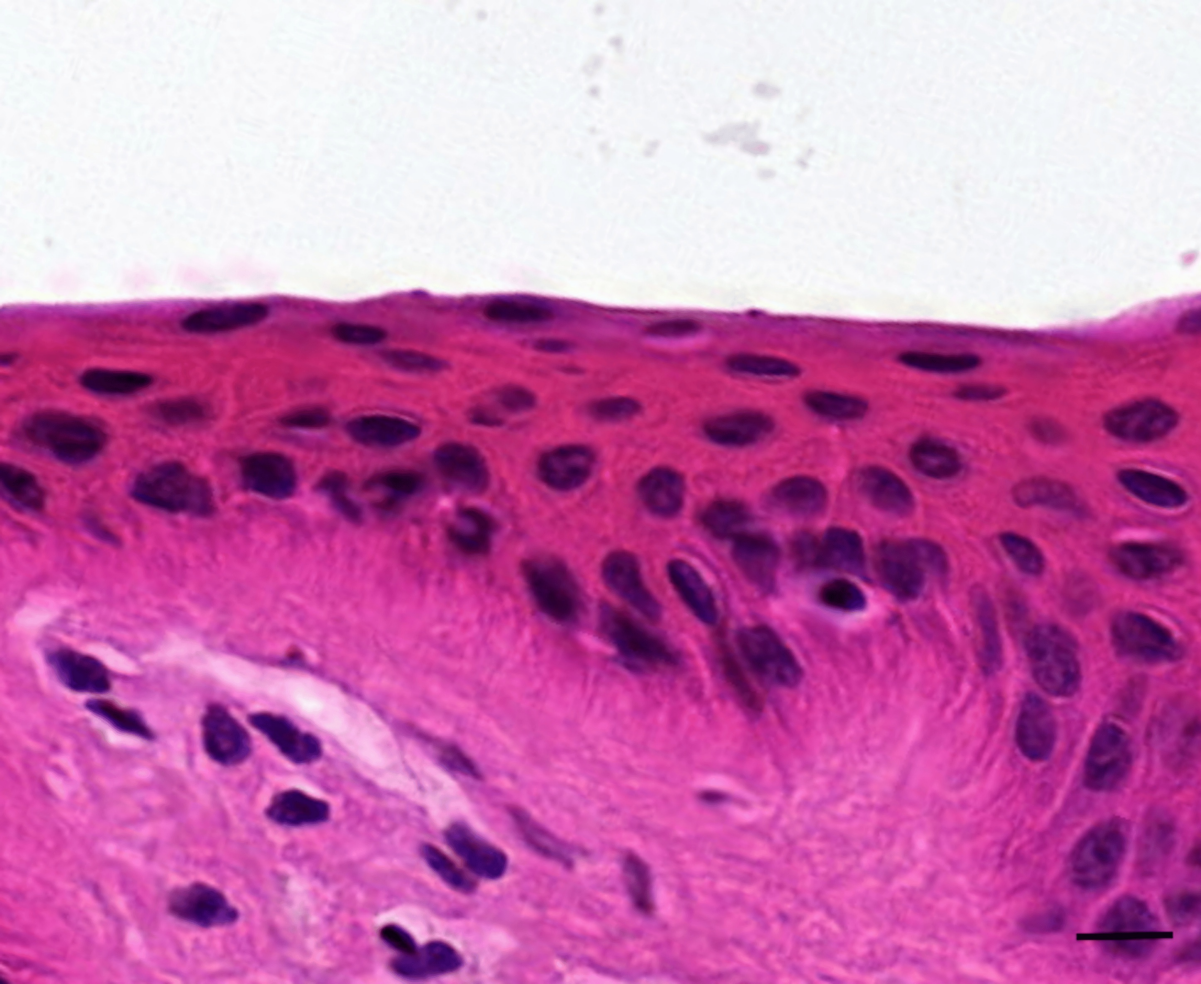
Histological slide of the skin after 40 tape strips with the stratum corneum completely removed (H&E stain) This sample was taken from the frozen-thawed porcine skin. The scale bar represents 10 µm. H&E: hematoxylin and eosin

For the fresh porcine, the initial thickness of the stratum corneum before tape application was determined to be 33.4 ± 2.0 µm, with only 7.1 ± 0.4 µm remaining after 20 strips, and the stratum corneum completely removed by strip 40. For the frozen-thawed porcine, the initial thickness of the stratum corneum before tape application was determined to be 31.0 ± 1.9 µm, with only 4.0 ± 1.3 µm remaining after 20 strips, and the stratum corneum also completely removed by strip 40. Table 1 shows the thicknesses of the stratum corneum for both the fresh and frozen-thawed porcine in relation to the number of tape strips performed. Figure 1 shows the histological slide image for the pre-stripped skin with full stratum corneum visible; this measurement was taken as a baseline stratum corneum thickness. Figure 2 shows the histological slide image for the skin after 40 tape strips with the stratum corneum completely removed.

**Table 1:** Number of tape strips versus SC thickness for fresh and frozen-thawed porcine skin from histological measurements SC: stratum corneum

Number of tape strips	Stratum corneum thickness in fresh porcine (µm)	Stratum corneum thickness in frozen-thawed porcine (µm)
0	33.4 ± 2.0	31.0 ± 1.9
1	28.1 ± 1.5	21.7 ± 0.9
2	27.1 ± 1.6	13.8 ± 1.4
3	26.0 ± 1.8	10.0 ± 1.1
4	23.7 ± 0.9	7.6 ± 0.6
5	22.2 ± 1.7	6.0 ± 0.9
10	17.5 ± 1.3	4.7 ± 1.0
20	7.1 ± 0.4	4.0 ± 1.3
40	0.0 ± 0.0	0.0 ± 0.0

**Figure 3:**
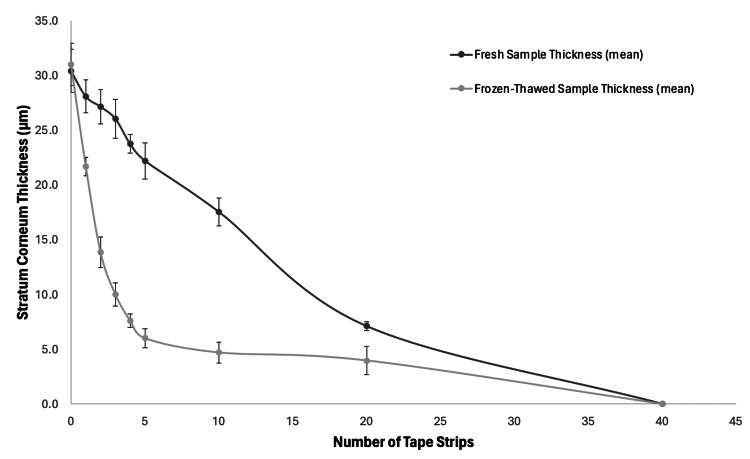
Graph of the stratum corneum thickness relative to the number of strips for both the fresh and frozen-thawed skin

For the fresh porcine, the stratum corneum thickness was correlated to the number of tape strips in a linear fashion (r = 0.96). For the frozen-thawed skin, the relationship was non-linear (r = 0.65). After the first five tape strips on the frozen-thawed skin, 80.6% of the stratum corneum had been removed compared to only 33.5% removed in the fresh porcine. Comparing the thickness of the fresh and frozen-thawed skin at each tape strip shows that they are statistically different (t(16) = 1.74, P = 0.03). The stratum corneum thickness relative to the number of strips for both the fresh and frozen-thawed skin is shown in Figure 3.

Difference in tape weight

**Figure 4:**
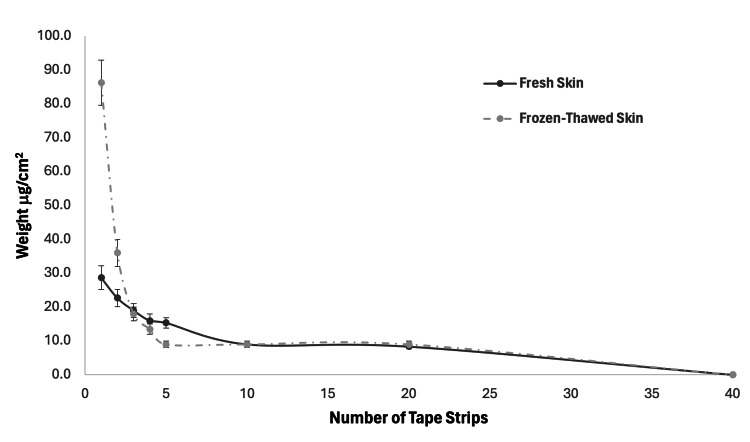
Graph of the weight of the cells removed by tape stripping for fresh and frozen-thawed porcine skin

The difference in weight results was normalized to µg/cm^2^. The first five tape strips showed the largest reduction of cells removed per strip. For the fresh porcine, strip one removed 28.7 ± 3.5 µg/cm^2^ of cells and strip two removed 22.7 ± 2.5 µg/cm^2^, meanwhile for the frozen-thawed skin, strip 1 removed 86.3 ± 6.7 20 µg/cm^2^, and strip 2 removed 36.0 ± 4.0 µg/cm^2^. However, for both the fresh and frozen-thawed specimens, strips 10-30 removed approximately 9.0 ± 1.0 µg/cm^2^ per strip consistently. Visually inspecting the tape after stripping using an LED bench light also gave an indication that the first five strips removed significantly more corneocyte cells than those applied after the first 10. Visually inspecting the tape also indicated more visible skin cells on the tape when stripping the frozen-thawed skin than the fresh. The non-linear removal of the stratum corneum by the tape on the frozen-thawed skin versus the more linear removal of the fresh skin is shown in Figure 4. Table 2 shows the weight of the skin cells removed in relation to the number of tape strips performed on the fresh and frozen-thawed skin.

**Table 2:** Weight of the skin cells removed in relation to the number of tape strips performed on the fresh and frozen-thawed skin

Tape strip number	Tape weight in fresh porcine (µg/cm^2^)	Tape weight in frozen-thawed porcine (µg/cm^2^)
1	28.7 ± 3.5	86.3 ± 6.7
2	22.7 ± 2.5	36.0 ± 4.0
3	19.0 ± 2.0	18.0 ± 1.1
4	16.0 ± 2.0	13.5 ± 1.5
5	15.3 ± 1.5	9.0 ± 1.0
10	9.0 ± 1.0	9.0 ± 1.0
20	8.3 ± 0.6	9.0 ± 1.0
40	0.0 ± 0.0	0.0 ± 0.0

Comparing the tape weighing method to the histological SC thickness measurements for both fresh and frozen-thawed samples using the Pearson correlation coefficient showed a strong correlation with r = 0.93 for the fresh samples. There was also a strong correlation between the two methods for the frozen-thawed samples with r = 0.95. 

## Discussion

This study aimed to examine the differences in behavior between fresh and frozen-thawed porcine skin during tape stripping experiments. Our findings highlight significant variations in the response of these two types of specimens, which carry important implications for dermatological and pharmacological skin research.

The data revealed that fresh and frozen-thawed porcine skin samples exhibit distinct patterns in response to tape stripping. Specifically, the stratum corneum (SC) of frozen-thawed skin was removed more rapidly compared to that of fresh skin. By the fifth tape strip, 80.6% of the SC was removed from frozen-thawed samples, whereas only 33.5% was removed from fresh samples. The removal of SC in fresh skin was correlated to tape strips in a linear pattern (r = 0.96), while in frozen-thawed skin, it exhibited a non-linear pattern (r = 0.65). These differences are statistically significant (P < 0.03) and suggest that the freezing and thawing processes alter the SC's mechanical integrity, leading to accelerated removal. This has implications for the interpretation of experimental results in studies assessing skin barrier function and topical treatments.

The tape weight measurements further support these findings. The first tape strip removed significantly more cells from frozen-thawed skin (86.3 ± 6.7 µg/cm²) compared to fresh skin (28.7 ± 3.5 µg/cm²). This pattern persisted for the initial strips, with the removal rate plateauing at approximately 9.0 ± 1.0 µg/cm² per strip for both types of skin after the 10th application. This rapid removal of cells from frozen-thawed skin likely indicates compromised SC integrity due to the freezing and thawing processes [22]. Visual inspection of the tape confirmed more visible corneocyte cells on initial strips with a reduction of cells on subsequent tape samples, aligning with previous studies that observed similar phenomena [7,9].

When comparing tape weighing to histological measurements, a strong correlation was observed for both fresh (r = 0.93) and frozen-thawed (r = 0.95) samples. This indicates that tape weighing is a reliable alternative to histological analysis for evaluating SC removal rates across both types of skin specimens, offering a more streamlined, faster, less resource-intensive, and less invasive method for assessing skin barrier function.

The limitations of this study include the findings being based on a limited sample size, which may affect the generalizability of the results. There will also be variability between skin sites on the porcine ears and between ear specimens, which may not have been accounted for given the small sample size. Additionally, the study's focus on tape weighing and histological analysis, while thorough, might benefit from incorporating other measurement techniques such as TEWL or infrared spectroscopy to provide a more comprehensive understanding of skin barrier function across the two sample types. Furthermore, the environmental conditions under which the samples were stored and tested could have introduced variability, and the impact of these conditions was not fully explored. Finally, while the study compared fresh and frozen-thawed samples, it did not investigate the effects of different freezing and thawing protocols, which could yield different results and implications for dermatological research.

The implications of these findings are multifaceted. In research applications, the choice between fresh and frozen-thawed specimens should be made based on this study's objectives. Fresh skin provides more predictable and linear removal patterns, ideal for experiments requiring precise control over SC removal. This is particularly relevant for studies on skin barrier function, drug penetration, and topical formulation efficacy [3-5].

## Conclusions

In conclusion, this study provides a detailed comparison of fresh and frozen-thawed porcine skin in tape stripping experiments, revealing significant differences in SC removal and highlighting the strong correlation between tape weighing and histological analysis. These findings are crucial for dermatological and pharmacological skin research, as they offer valuable insights for optimizing experimental designs and improving the interpretation of results. By accounting for specimen conditions and standardizing methodologies, researchers can advance dermatological research and enhance patient care.
